# The Wafold: Curvature-Driven Termination and Dimensional Compression in Black Holes

**DOI:** 10.3390/e28010022

**Published:** 2025-12-24

**Authors:** Javier Viaña

**Affiliations:** Department of Physics and Kavli Institute for Astrophysics and Space Research, Massachusetts Institute of Technology, Cambridge, MA 02139, USA; vianajr@mit.edu; Tel.: +1-513-356-2584

**Keywords:** black holes, event horizon, dimensional transition, holographic scaling, gravitational entropy

## Abstract

This work explores a geometric description of black holes in which spacetime terminates on a curvature-triggered hypersurface rather than extending to an interior singularity. We study the implications of a scenario in which, upon reaching a critical curvature threshold, the three-dimensional spatial geometry compresses into a thin, closed boundary identified here as the *wafold*. Beyond this, the manifold would no longer continue, and all mass–energy and information would be confined to the hypersurface itself. This framework combines two well-explored paths: (1) curvature-driven geometric compression, in which extreme curvature forces the bulk degrees of freedom to become supported on a thin hypersurface (without altering the underlying dimensionality of spacetime), and (2) the motivation underlying the holographic principle, namely that black-hole entropy scales with surface area rather than volume, suggesting that information is governed by a boundary geometry rather than a bulk volume. We elaborate a dimensional conversion law that would be required to describe the collapse of spatial volume into surface area as a conserved flux of geometric capacity across the wafold, and we analyze the resulting consequences of treating this hypersurface as the terminal boundary of the manifold.

## 1. Introduction

Black holes remain among the most profound predictions of Einstein’s general relativity (GR), combining the elegance of geometric simplicity with the challenge of physical incompleteness. Classical GR predicts that the endpoint of gravitational collapse is a spacetime singularity, where curvature invariants diverge and the manifold description fails [1,2]. Such divergences are generally interpreted as indicators that the classical field theory has exceeded its domain of validity, demanding new geometric or quantum principles to describe the high-curvature regime.

A vast body of work has attempted to reconcile these inconsistencies, ranging from quantum-gravity inspired models [3,4,5,6,7] to string-theoretic microstate constructions [8,9]. Yet all such efforts confront the same geometric challenge: how to remove the singularity while preserving the empirically verified exterior spacetime. The external Schwarzschild or Kerr geometries remain among the best-tested predictions of GR, confirmed through gravitational-wave ringdowns, black-hole shadows, and orbital dynamics [10,11,12]. Any viable modification must therefore reproduce these external metrics exactly, while addressing the breakdown of the manifold at the center.

In this theory, we examine a framework in which spacetime terminates once the curvature reaches a critical threshold, thereby avoiding the formation of a singularity. We study the possibility that the collapsing domain reorganizes into a thin, curved hypersurface, that we identified as the *wafold*, onto which all infalling mass–energy becomes confined. This layer represents a geometric transition, a change in how degrees of freedom are supported, concentrating them onto a hypersurface while preserving the four-dimensional structure of spacetime.

This modification concerns only the deepest layer of collapse, replacing the classical singularity. The region between the event horizon and the terminating surface remains, as in standard GR, inaccessible to external observers. Accordingly, the causal structure is unchanged: nothing inside the horizon can transmit information outward. Because the manifold ends at this curvature-defined surface, there is no interior domain into which classical or quantum trajectories could extend; propagation is defined only on the exterior side of the hypersurface.

The proposal connects to several established lines of research. The first is the holographic scaling of entropy. The Bekenstein–Hawking relation, $S=k_{B}A/4L_{P}^{2}$ [13,14], implies that all information associated with a black hole scales with surface area rather than volume. This inspired the *holographic principle* [15,16,17,18], according to which the complete information content of a gravitational region may be captured by fields defined on a boundary of lower dimension than the bulk. In the present theory, we take a different geometric direction for implementing an area-based description: as collapse proceeds, the wafold is the sole physical carrier of the system’s degrees of freedom. Unlike holographic dualities, which describe an existing spacetime region related to a surface, the wafold posits that the bulk itself never forms; the boundary is all that remains of spacetime.

A second connection arises with thin-shell and surface-layer formalisms. The wafold can be represented as a localized geometric surface embedded within a classical spacetime, analogous to constructions in the Israel junction framework [19,20]. This perspective resonates with several horizon-scale alternatives such as *gravastars* [21,22] and other compact surface or regularity-based models [23,24,25], where the interior is replaced by a de Sitter core and a thin shell of matter that matches to the external Schwarzschild geometry. In those models, collapse comes to a geometric limit through a transition between positive and negative pressure regions. However, such scenarios require specific matter equations of state and finely tuned shell properties to remain stable. By contrast, the wafold does not depend on any specific matter content: it is a geometric termination of the manifold itself, a hypersurface where spacetime ends and matter becomes confined. In this preliminary picture it need not be supported by conventional pressure or tension; rather, its role is geometric.

At the microscopic end of the theoretical spectrum, the *fuzzball* proposal in string theory [8,9] replaces the classical interior with a vast ensemble of horizonless microstate geometries, each resolving the singularity at the Planck scale. In this case, the information is not carried by a vacuum interior, but by an intricate configuration of strings and branes that fills the region which would classically lie inside the event horizon. In this view, each black-hole microstate corresponds to a specific, smooth geometry whose structure encodes the information of the underlying quantum state. The exterior region matches the usual black-hole metric, but instead of an empty interior ending at a singularity, the geometry transitions into a complicated, nonsingular structure supported by string and brane excitations. Different quantum microstates correspond to different versions of this internal structure, and in certain supersymmetric cases the number of known solutions reproduces the expected Bekenstein–Hawking entropy. This approach provides a concrete microscopic picture of information storage, but its explicit realizations require strong supersymmetry, special charge assignments, and controlled internal manifolds. As a result, fuzzball geometries are known mainly for extremal or near extremal objects, while the generic non-extremal, rotating, dynamically formed black holes relevant for astrophysics remain out of reach. A complete fuzzball description of realistic collapse or evaporation is still an open problem.

The wafold framework approaches the same question from a purely geometric and macroscopic angle. Rather than replacing the interior with detailed microstate geometries, it assumes that spacetime terminates at the surface where curvature reaches a universal threshold. All mass, energy, and information are confined to this terminal hypersurface, which acts as the geometric endpoint of the manifold. The exterior geometry remains the classical Schwarzschild or Kerr solution, while the interior region never forms. In contrast to fuzzballs, which maintain a complicated and extended internal structure, in the wafold proposal, the interior collapses into a single geometric layer. Thus, while both frameworks eliminate the singularity and prevent information from disappearing into a classical interior, the fuzzball replaces that interior with a microscopic horizonless geometry, whereas the wafold replaces it with a true termination of spacetime determined by a curvature limit. Other microscopic perspectives, such as the quantum N-portrait [26], likewise aim to replace the classical interior with quantum structure, but differ in mechanism and ontology.

Related in spirit, but physically distinct, are the *firewall* proposals [27,28,29] that posit a breakdown of quantum entanglement at or near the event horizon. In firewall scenarios, infalling observers encounter a high-energy barrier that disrupts smooth horizon crossing, resolving the information paradox through quantum-mechanical nonlocality. Yet even here, spacetime continues beyond the firewall; it is the quantum state, not the manifold, that is discontinuous.

Another approach to singularity resolution arises in *Planck star* models [4,30,31], where quantum-gravity pressure arrests collapse at Planck density and triggers a subsequent expansion or bounce. In those models, the black-hole interior survives as a high-density core that eventually tunnels into a white hole. The wafold departs sharply from this picture: no bounce, interior, or continued evolution is assumed.

Finally, the conceptual origin of the present theory aligns with emergent-spacetime frameworks, where the effective properties of geometry depend on curvature or energy scale [32,33]. The wafold can be viewed as a macroscopic manifestation of this idea: when curvature exceeds a critical value, the spatial geometry undergoes a dimensional compression, conserving total information while preventing further geometric evolution.

Taken together, these diverse frameworks, from holography to gravastars, fuzzballs, firewalls, and Planck stars, share a common motivation: to replace the classical singularity with a finite, information-preserving structure. Yet they differ fundamentally in their mechanisms. Gravastars rely on exotic matter; fuzzballs on microscopic string geometries; firewalls on quantum entanglement breakdown; and Planck stars on quantum gravitational pressure. On the other hand, the wafold introduces no new matter content, quantum degrees of freedom, or exotic equations of state. It is a purely geometric proposal: a curvature-triggered termination of spacetime in which the interior manifold never forms.

Concepts in which spacetime itself terminates also appear in other contexts. In Witten’s “bubble of nothing” [34], a compact dimension collapses smoothly and the manifold ends on an expanding boundary beyond which no geometry exists. Similarly, end-of-the-world brane constructions in AdS/BCFT frameworks [35,36] describe spacetimes that terminate on codimension-one hypersurfaces carrying the boundary degrees of freedom. The Hartle–Hawking no-boundary proposal [37] and certain AdS wormhole geometries [38] likewise illustrate how the manifold can end smoothly without singularities. Dimensional-reduction models in quantum gravity [39] further suggest that at high curvature, the effective dimensionality of spacetime itself may collapse, echoing the geometric compression envisioned here. These frameworks collectively anticipate the possibility that spacetime may possess a genuine geometric endpoint rather than an interior continuation.

While these ideas illustrate that spacetime termination can occur in diverse settings, none provides a fully geometric account of a curvature-driven collapse of the manifold itself into a hypersurface that confines all mass–energy and information inside the black hole. Closely related frameworks (such as the membrane paradigm [40] or holographic interpretations [41]) treat the horizon as an effective surface carrying these properties, but they do not identify a curvature-set terminal surface as the literal end of spacetime. The wafold hypothesis therefore represents a novel geometric alternative to both matter-supported and microstate-based models, one that preserves known exterior predictions of general relativity while replacing the unobservable interior with a causally complete, information-conserving boundary.

This work is presented as an exploratory alternative, aimed at motivating further mathematical, numerical, and physical work on entropy, information conservation, and boundary geometry in gravitational collapse. Throughout, we distinguish the wafold from global and quasi-local horizons (event, apparent, or trapping): the wafold is defined by a critical-curvature condition and therefore need not coincide with any of these, especially in dynamical or highly massive black holes [42,43].

## 2. The Wafold

### 2.1. Theory Overview

Before introducing the formal construction, we briefly outline the underlying goal. In classical general relativity, continued gravitational collapse inside a black hole leads inevitably to a curvature singularity, where the geometric invariants of the spacetime diverge and the classical description ceases to be meaningful. Many approaches to quantum gravity suggest that such divergences should be resolved or avoided by new geometric behavior at an extremely high curvature. With this expectation in mind, we examine whether a purely geometric mechanism based on a limiting curvature could prevent the formation of singularities within the classical framework itself.

We begin by exploring the hypothesis that curvature may possess a fundamental upper limit, as suggested in various quantum-gravity-inspired models. In this perspective, spacetime can sustain curvature only up to a threshold; once that limit is reached, the classical geometric description cannot accommodate any further curvature growth. Consequently, when gravitational collapse pushes a region toward this bound, the interior geometry must adopt a different configuration. To remain consistent with the exterior Schwarzschild solution and with regularity conditions, we propose that this transition manifests as a reorganization of the collapsing spatial region: instead of continuing to contract, the three-dimensional interior compresses into a closed, thin, shell-like hypersurface located precisely at the locus where curvature saturates its limit. We refer to this structure as the wafold, from “wall that folds”, denoted by W. In subsequent sections, we analyze the mathematical consistency of this transition and its implications for avoiding singularity formation.

From the exterior perspective, the process may appear as the emergence of a bubble-like layer that absorbs the collapsing mass onto its surface. All mass–energy and information that would otherwise reside within the interior become confined to the wafold.

To formalize this hypothesis, we introduce a curvature threshold $K_{c}$ above which the classical manifold description cannot be sustained. A curvature trigger must be formulated as a scalar invariant of the metric, ensuring that the threshold condition $\leq K_{c}$ is coordinate independent. Several natural curvature scalars might in principle serve this role. A first possibility is the Ricci scalar *R*. However, in all vacuum black-hole spacetimes (Schwarzschild and Kerr) one has R=0 everywhere outside matter sources. It therefore carries no information about the growth of tidal curvature in vacuum and cannot identify the onset of gravitational collapse. The same issue affects the Ricci contraction $R_{\mu \nu }R^{\mu \nu }$, which also vanishes identically in vacuum. These Ricci-based invariants are therefore blind to the very curvature that becomes extreme near the classical singularity.

Another possibility is to use the square of the Weyl tensor, $C_{\mu \nu \rho \sigma }C^{\mu \nu \rho \sigma }$, which measures the tidal part of the gravitational field and does diverge at the Schwarzschild singularity. However, in vacuum spacetimes the Riemann tensor decomposes into the Weyl tensor alone, so that $R_{\mu \nu \rho \sigma }R^{\mu \nu \rho \sigma }=C_{\mu \nu \rho \sigma }C^{\mu \nu \rho \sigma }$, making the Weyl invariant equivalent to the full Riemann-square invariant. Using the Weyl scalar therefore offers no additional discriminatory power and yields precisely the same threshold condition as the Riemann-square approach.

Having excluded the Ricci scalar and the Ricci contraction, and noting the equivalence of the Weyl and Riemann invariants in the vacuum region relevant to black holes, the only curvature scalar that remains both nontrivial in vacuum and sensitive to the divergence at the classical singularity is the Kretschmann scalar, $K\equiv R_{\mu \nu \rho \sigma }R^{\mu \nu \rho \sigma }$. It remains finite at the horizon, diverges at the singularity, and provides a clean, invariant measure of curvature growth during collapse. For these reasons we adopt *K* as the trigger and impose the limiting-curvature condition

(1) $$K\left(x\right)\leq K_{c},$$

for all points *x* in physical spacetime.

On dimensional grounds, one would expect any universal upper bound for the Kretschmann scalar to be of the order of the Planck curvature, $K_{c}∼L_{P}^{-4}$, as this is the scale at which quantum-gravity effects are generally anticipated. We therefore regard $K_{c}$ as a parameter of Planckian size whose precise value would ultimately be fixed by a full quantum-gravity theory, requiring that it be sufficiently large to preserve the well-tested exterior geometry of astrophysical black holes.

Accordingly, classical general relativity remains valid whenever $K\ll K_{c}$. As *K* approaches $K_{c}$, the geometry can no longer evolve toward a higher curvature, and at $K=K_{c}$ the spacetime terminates on the wafold hypersurface.

In a Schwarzschild spacetime of mass *M* (with G=c=1),

(2) $$ds^{2}=-\left(1-\frac{2M}{r}\right)dt^{2}+{\left(1-\frac{2M}{r}\right)}^{-1}dr^{2}+r^{2}d\Omega^{2},$$

the Kretschmann scalar is

(3) $$K\left(r\right)=\frac{48M^{2}}{r^{6}}.$$

The wafold radius $r_{W}$ is therefore determined by

(4) $$K\left(r_{W}\right)=K_{c},$$

which gives

(5) $$r_{W}={\left(\frac{48M^{2}}{K_{c}}\right)}^{1/6}={\left(\frac{48}{K_{c}}\right)}^{1/6}M^{1/3}.$$

Hence, for a fixed universal threshold $K_{c}$, the wafold radius scales as $r_{W}\propto M^{1/3}$, whereas the Schwarzschild radius scales as $r_{s}=2M$. Their ratio is

(6) $$\frac{r_{W}}{r_{s}}={\left(\frac{3}{4K_{c}}\right)}^{1/6}M^{-2/3}.$$

**Figure 1:**
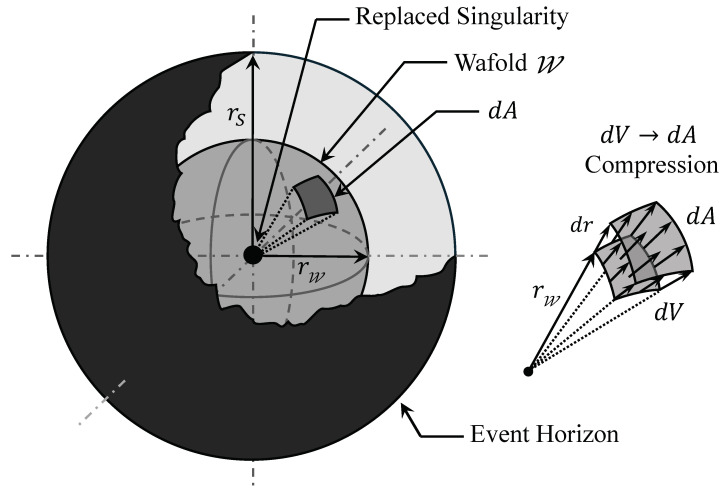
Schematic representation of a Schwarzschild wafold W with radius $r_{W}$. The collapsing interior volume dV is compressed into a surface element dA, replacing the classical singularity inside the event horizon.

For astrophysical black holes with $M\gg M_{P}$, this ratio is extremely small: $r_{W}$ lies deep inside the event horizon, replacing only the classical singularity while leaving the exterior geometry unchanged. As *M* approaches the Planck mass, $r_{W}$ moves outward toward $r_{s}$, and the resulting black-hole configuration becomes increasingly dominated by the wafold, consistent with the expectation that near-Planckian black holes have no classical interior. In Section 2.4, we will show that this curvature-defined radius provides a consistent location for the terminal surface in a spherically symmetric construction. A representation of the interior’s geometric collapse for the spherically symmetric case is illustrated schematically in Figure 1.

The wafold therefore represents a curvature-triggered terminal surface of the spacetime manifold. To express this rigorously, let $\left(M,g_{\mu \nu }\right)$ denote a four-dimensional, smooth, time-oriented Lorentzian spacetime manifold, and let W be a closed, embedded codimension-one hypersurface on which spacetime terminates. We treat W as the boundary of M in the geometric sense,

∂M=W,

meaning that the manifold is defined only on one side of W and admits no smooth extension of the metric through the hypersurface. In high-energy language, such a one-sided terminal hypersurface is an *end-of-the-world brane*: a surface carrying the induced geometry, beyond which no spacetime exists.

The following formulation helps clarify the intuitive distinction between the region where spacetime is defined and the domain beyond it. We define the *reality* region as

R:=M,

the domain in which the metric, curvature, causal structure, and geodesics are well defined. To express the complement of the physical spacetime, we introduce the notion of a “non-reality’’ region. This is not a second geometric domain but simply

NR:=⌀,

indicating the absence of any differentiable structure beyond W. What would have constituted an interior region is instead geometrically collapsed onto W: all degrees of freedom that would have resided inside are now supported on the terminal hypersurface itself.

Because spacetime ends at W, only limits taken from within *R* exist:

$$\exists \lim_{x\to W^{+}}g_{\mu \nu }\left(x\right)=g_{\mu \nu }^{\left(+\right)},\;\;∄\lim_{x\to W^{-}}g_{\mu \nu }\left(x\right).$$

Here, the superscript (+) denotes approach from the exterior of the wafold (the side corresponding to physical reality), whereas (−) refers to approach from the interior, the non-real side.

All geometric quantities on W are therefore defined solely through this exterior approach. In this mathematical sense, the wafold is the *terminal boundary* of spacetime: the unique hypersurface at which the Lorentzian manifold ends. It is not a thin shell separating two regions, nor a distributional hypersurface across which the metric continues, but a genuine geometric endpoint of the spacetime manifold.

### 2.2. Geometry of the Terminal Hypersurface

We define geometric quantities as positive in the direction of the outward unit normal $n^{\mu }$, oriented from W toward the domain of reality. With this orientation, $n_{\mu }n^{\mu }=\varepsilon$, and ε±1, where ε=+1 corresponds to a space-like normal and ε=−1 to a time-like one. Null segments will require a separate treatment. The intrinsic and extrinsic geometry of W, defined with respect to the exterior manifold, are then

(7) $$h_{\mu \nu }=g_{\mu \nu }^{\left(+\right)}-\varepsilon n_{\mu }n_{\nu },\;\;K_{\mu \nu }^{\left(+\right)}={h_{\mu }}^{\alpha }{h_{\nu }}^{\beta }\nabla_{\alpha }^{\left(+\right)}n_{\beta }.$$

Here, $h_{\mu \nu }$ is the metric induced on W, and $K_{\mu \nu }^{\left(+\right)}$ is the corresponding one-sided spacetime extrinsic curvature, computed using the Levi–Civita connection $\nabla^{\left(+\right)}$ of the exterior metric $g_{\mu \nu }^{\left(+\right)}$. Since the manifold terminates at W and has no extension in the $-n^{\mu }$ direction, the extrinsic curvature is defined only on the physical side ($K_{\mu \nu }^{\left(+\right)}$), describing how the final layer of spacetime bends when approached from the exterior.

For quantities defined on W, it is convenient to project the spacetime extrinsic curvature onto directions tangent to the surface. The resulting surface-projected extrinsic curvature $K_{ab}^{\left(+\right)}$ is given by ${e_{a}}^{\mu }{e_{b}}^{\nu }K_{\mu \nu }^{\left(+\right)}$, and captures the local geometric shape of the wafold as seen from within the surface itself. Because the manifold terminates at W, only the exterior limit of the metric and its induced geometric structures are well defined. No extension of geodesics or fields exists across the hypersurface; therefore, no geometric quantities can be assigned to any “interior” side. All physically meaningful structures on W (such as proper time, distances, and tangential derivatives) arise from the induced metric $h_{ab}$ and the one-sided surface-projected extrinsic curvature $K_{ab}^{\left(+\right)}$. The wafold therefore behaves as a geometric boundary: all operationally definable quantities end on W.

We do not define an interior region, because in the wafold construction no such region exists: the manifold is defined only up to the terminal hypersurface W and admits no extension for $r<r_{W}$.

All geometric and field-theoretic data are therefore understood as exterior limits approaching W. The dynamical role of the terminal hypersurface can be consistently formulated within this one-sided framework by treating W as a boundary of the spacetime manifold. In Section 2.3, we develop this formulation and elaborate its dynamical implications for gravitational collapse. This structure is implemented through a one-sided variational principle supplemented by the required boundary term, which encodes the effective surface stress–energy associated with the wafold [44,45].

With this interpretation, W is a genuine geometric endpoint: the would-be interior $r<r_{W}$ is absent from the differentiable structure, so neither classical geodesics nor quantum field modes have a domain on which they could be continued beyond W. In particular, quantum particles (understood as excitations of fields defined on the spacetime manifold) cannot “enter” the non-reality region, because no such region exists for their underlying fields to propagate into. No additional physical mechanism is required to “prevent” crossing; propagation simply ceases to be defined once the manifold terminates. This behavior is directly analogous to end-of-the-world branes in AdS/BCFT or to Witten’s bubble of nothing, where both classical trajectories and quantum excitations end at a one-sided boundary. In this sense, W functions as the terminal boundary of the physical spacetime, not as a barrier within it.

### 2.3. Dynamical Origin of the Wafold

As *K* approaches the critical value $K_{c}$, tidal distortions per unit length become comparable to the inverse square of the shortest physically meaningful length scale. This marks the regime in which we identify a breakdown of volumetric coherence, understood here as the ability of finite spatial volume elements to maintain three-dimensional integrity under tidal deformation. Beyond this point, spacetime can no longer realize such regions as extended manifold domains. Dynamically, the proposed system resolves this instability by reorganizing the collapsing region into the lowest-dimensional structure capable of supporting the required curvature: a thin hypersurface. This is a familiar phenomenon in elasticity and geometry: when a bulk medium is overstrained, it relieves stress by localizing deformation onto lower-dimensional folds or sheets. The present theory is the relativistic analogue of this phenomenon.

The wafold is based on a single physical hypothesis: a fundamental upper bound on spacetime curvature, $K\leq K_{c}$. Wherever this bound is not saturated, the classical description of spacetime is assumed to remain valid. In those regions, consistency with general relativity and with observations requires the metric to coincide with the Schwarzschild/Kerr solution. The next question is dynamical: what completion of collapse is compatible to preserve exact exterior Schwarzschild/Kerr invariance? We argue that, under these two requirements alone (curvature limit and exterior consistency), the curvature cap is dynamically sufficient to force a specific transformation of the collapsing interior. Below, we discuss why the minimal forced completion is the wafold configuration: the interior ceases to exist as a physical manifold, and the conserved content is encoded as surface data on the terminal hypersurface W defined by $K=K_{c}$.

The argument begins with a bookkeeping fact. For an exterior observer, the total ADM mass *M* is fixed by the asymptotic geometry and cannot disappear. Once matter crosses the horizon, classical re-emergence is not possible; therefore, it must be supported either (a) in an interior region (if it exists), (b) on a boundary layer, or (c) in some mixture of both. The curvature cap, together with exterior invariance, sharply restricts these possibilities.

Why is it that an interior region cannot generically store the mass under a hard cap? Assume, for contradiction, that there exists a genuine interior manifold region Z extending to radii $r<r_{W}$ while the exterior remains exactly Schwarzschild. Consider any world tube of infalling matter that continues into Z. If the matter can proceed into $r<r_{W}$, then either (i) the geometry in Z remains close to Schwarzschild, in which case the curvature necessarily exceeds $K_{c}$ and the cap is violated, or (ii) the geometry in Z departs from Schwarzschild so as to keep $K\leq K_{c}$. Option (ii) necessarily requires a nontrivial interior stress–energy that dynamically prevents further infall into the forbidden region. Crucially, the curvature cap itself cannot supply such a mechanism if Z exists as a manifold: a bound on a scalar invariant does not generate a force law or boundary condition capable of arresting geodesics. Preventing matter from entering $r<r_{W}$ while preserving an exact Schwarzschild/Kerr exterior therefore requires introducing additional structure beyond the two postulates above, such as a material barrier (pressure or tension layer), a reflective boundary condition, a bounce supported by NEC-violating effective stress–energy, or a regular core with a new length scale and finely tuned junction conditions. In all those cases, the completion relies on extra ingredients not implied by exterior invariance plus the curvature bound.

There is, however, one completion in which the curvature cap is sufficient by itself. If spacetime is taken to terminate at the saturation locus W, then no additional mechanism is required to prevent further infall: matter cannot proceed into $r<r_{W}$ simply because no spacetime exists there. Under this completion, the curvature cap enforces the transformation directly, while exterior Schwarzschild/Kerr invariance is preserved automatically. In this case, the conserved content that has crossed the horizon must be supported on W itself. This is the wafold completion. It replaces the need for an internal stopping force or repulsive core with a geometric endpoint dictated solely by the curvature bound and exterior invariance.

With no interior manifold available beyond W, collapse admits only a singular limit in which the interior support shrinks to zero thickness at the saturation locus. Therefore, a compression is the unavoidable geometric continuation of collapse once spacetime termination is imposed.

More formally, once collapse is completed by termination at W, the gravitational content associated with the fixed exterior geometry may be described as data localized on that surface. In general relativity, the natural framework for such localization is provided by surface-supported stress–energy. This description can be obtained as the limit of a family of smooth collapsing configurations in which stress–energy and curvature are concentrated in a layer of finite thickness near the saturation locus. Let ϵ denote the proper thickness of this layer. A compression process is then modeled by taking ϵ→0 while the total mass measured at infinity remains *M*. In this limit, the stress–energy tensor $T_{\mu \nu }^{\left(\epsilon \right)}$ converges, in the distributional sense, to a decomposition into a regular bulk contribution and a surface-supported term,

(8) $$T_{\mu \nu }^{\left(\epsilon \right)}⟶T_{\mu \nu }^{\left(\mathrm{reg}\right)}+S_{\mu \nu }\delta \left(\ell \right),$$

where *ℓ* is the Gaussian normal distance to W and δ(ℓ) denotes the Dirac delta distribution with support on the hypersurface W. The tensor $T_{\mu \nu }^{\left(\mathrm{reg}\right)}$ represents any stress–energy that remains finite and nonsingular away from W, while $S_{\mu \nu }$ encodes the finite energy–momentum density localized on the terminal hypersurface.

In the wafold completion considered here, spacetime terminates at W, so no bulk region remains to support stress–energy. Accordingly, the regular contribution $T_{\mu \nu }^{\left(\mathrm{reg}\right)}$ vanishes, and the thin-layer limit reduces to a purely surface-supported distribution,

(9) $$T_{\mu \nu }^{\left(\epsilon \right)}⟶S_{\mu \nu }\delta \left(\ell \right),$$

again with *ℓ* denoting the Gaussian normal distance to W. Equations (8) and (9) provide a precise mathematical meaning of compression: the support of the collapsing interior shrinks in the normal direction while its integrated gravitational content is preserved. Physically, the limit ϵ→0 is an idealization; the resulting wafold is expected to retain a minimal physical thickness set by the shortest meaningful length scale, plausibly of the order of the Planck length.

This thin-layer limit is not an additional assumption; it is the standard weak completion underlying thin-shell gravity. It supplies exactly what alternative interior completions must introduce by hand. Here, the curvature cap alone is sufficient to drive the transformation, because the termination of spacetime removes the need for any additional barrier or interior dynamics.

Once spacetime terminates at W, the surface data are fixed by the exterior geometry through a one-sided variational principle. Accordingly, the Einstein–Hilbert action on the admissible domain must be supplemented by a one-sided Gibbons–Hawking–York boundary term on W, leading to

(10) $$S\left[g\right]=\frac{1}{16\pi }\int_{r\geq r_{W}}R\sqrt{-g}d^{4}x+\frac{1}{8\pi }\int_{W}K^{\left(+\right)}\sqrt{\left|h\right|}d^{3}x+S_{\mathrm{ext}}.$$

Here, *g* and *h* denote the determinants of the exterior spacetime metric and the induced metric on W, respectively; *R* is the corresponding Ricci scalar; $K^{\left(+\right)}\equiv h^{ab}K_{ab}^{\left(+\right)}$ is the trace of the one-sided extrinsic curvature of W; and $S_{\mathrm{ext}}$ accounts for any additional matter or flux contributions in the exterior region.

With this one-sided action in place, variation with respect to the induced metric $h_{ab}$ on W yields the associated surface stress–energy tensor,

(11) $$S_{ab}=\frac{1}{8\pi }\left(K_{ab}^{\left(+\right)}-h_{ab}K^{\left(+\right)}\right),$$

which is the one-sided Brown–York stress tensor. Because no interior region exists, W is not a junction surface and no matching conditions are imposed. Therefore, the resulting relation between the extrinsic curvature and the intrinsic surface stress tensor coincides with the one-sided limit of the Israel junction condition.

In summary, the curvature cap guarantees a saturation locus $K=K_{c}$. When exact exterior Schwarzschild/Kerr invariance is imposed, this cap is sufficient to force a transformation of the collapsing interior. Any completion that retains an interior manifold requires additional structure to prevent matter from entering the forbidden region and therefore goes beyond the minimal assumptions. The wafold completion avoids this: spacetime terminates at W, the curvature bound is respected without introducing extra dynamics, and the conserved content is encoded as surface data fixed by the exterior geometry. In this sense, dimensional compression is the minimal geometric completion of curvature-limited collapse compatible with exterior invariance.

### 2.4. Spherically Symmetric Schwarzschild Wafold

To make the previous discussion more concrete, we elaborate on the spherically symmetric toy model. We take the exterior region to be the Schwarzschild solution introduced earlier, and we place the terminal surface at the curvature-saturating radius $r_{W}$ given by Equation (5). No interior spacetime is assumed for $r<r_{W}$; the manifold simply ends there. The corresponding radial domains of the spacetime are summarized in Table 1. For exterior observers, all rows below the horizon remain causally hidden; the subdivision of the interior is only a theoretical description of what infalling observers would traverse.

At $r=r_{W}$, the induced geometry follows immediately from the exterior metric. Restricting the Schwarzschild line element to this surface gives

$$h_{ab}dx^{a}dx^{b}=-\left(1-\frac{2M}{r_{W}}\right)dt^{2}+r_{W}^{2}\left(d\theta^{2}+\sin^{2}\theta d\phi^{2}\right),$$

in agreement with the general expression $h_{\mu \nu }=g_{\mu \nu }^{\left(+\right)}-\varepsilon n_{\mu }n_{\nu }$ introduced in Equation (7).

Because the spacetime exists only for $r>r_{W}$, the extrinsic curvature is computed solely from the exterior. Using an outward-pointing normal, one obtains

$${K^{(+)\theta }}_{\theta }={K^{(+)\phi }}_{\phi }=\frac{\sqrt{\left|1-\frac{2M}{r_{W}}\right|}}{r_{W}},\;\;{K^{(+)t}}_{t}=\frac{M}{r_{W}^{2}\sqrt{\left|1-\frac{2M}{r_{W}}\right|}},$$

where the absolute value compensates for the interior signature flip, since the wafold always lies inside the horizon. These follow directly from the one-sided definition of Equation (7). The associated surface stress tensor is given by the one-sided Brown–York expression (11), evaluated as an exterior limit on W.

The Schwarzschild wafold radius tracks the limiting-curvature locus defined by the saturation of the Kretschmann scalar,

$$r_{W}\left(t\right)={\left(\frac{48M{\left(t\right)}^{2}}{K_{c}}\right)}^{1/6},\;\;{\dot{r}}_{W}\left(t\right)=\frac{1}{3}\frac{r_{W}\left(t\right)}{M(t)}\dot{M}\left(t\right).$$

In the static case, it lies at a constant radius inside the horizon. Since the normal direction to a surface of fixed *r* is time-like for r<2M, the hypersurface W is space-like. In dynamical situations, its trajectory $r_{W}\left(t\right)$ may change the sign of $n_{\mu }n^{\mu }$, such as in evaporation, allowing W to become time-like depending on the evolution of $r_{W}\left(t\right)$.

### 2.5. Rotating Wafold and Jet Formation

Astrophysical black holes frequently produce powerful, highly collimated jets emerging along their rotation axes, ejecting large amounts of energy and angular momentum into interstellar space. The physical origin of these jets remains an active area of research [46]; in conventional models, they are attributed to magnetohydrodynamic processes in which spacetime rotation couples to magnetic fields near the event horizon [47,48,49,50].

A rotating wafold provides a distinct geometric mechanism for jet formation. Because the spacetime manifold terminates at the curvature-saturated hypersurface W, all mass–energy, surface currents, and any trapped radiation reside on this boundary rather than within a hidden interior. The relevant energy and momentum fluxes in the exterior region are encoded in the one-sided stress–energy tensor $T_{\mu \nu }^{\left(+\right)}$, which governs how matter and radiation interact with the geometry outside the wafold.

In a Kerr-like exterior, the rotational strain induced on W is maximal near the equator and minimal near the poles, where the horizon angular velocity $\Omega_{H}$ vanishes. The polar regions therefore represent directions of minimal rotational resistance: the centrifugal barrier is weakest, frame dragging is smallest, and electromagnetic fields naturally align with the rotation axis. As a consequence, surface-bound energy (whether supplied externally by accretion or generated internally by stresses or semiclassical dissipation) tends to migrate toward the poles, where escape into the exterior spacetime becomes dynamically favored. In this view, jet collimation arises directly from the geometry of the rotating terminal surface, rather than from magnetohydrodynamic effects anchored to an event horizon.

Angular-momentum conservation reinforces this surface-to-axis drainage. Observationally, astrophysical systems show a strong empirical connection between black-hole spin and jet power, with rapidly rotating objects tending to host the most luminous and collimated outflows [51,52,53]. In the present framework, this behavior can be linked to a standard geometric identity: in any spacetime with an axial Killing vector $\phi^{\mu }$, the flux of angular momentum across a surface is given by the Killing-current contraction $T_{\mu \nu }^{\left(+\right)}\phi^{\mu }n^{\nu }$. Integrating this flux over the polar caps of the wafold yields

(12) $$\frac{dJ}{dt}=-\int_{P}T_{\mu \nu }^{\left(+\right)}\phi^{\mu }n^{\nu }dA,$$

where $n^{\nu }$ is the outward unit normal to P and P denotes the polar regions of the wafold. The minus sign reflects the convention that positive flux through P corresponds to angular momentum leaving the system. Radiation escaping through the poles therefore not only releases energy but also spins down the rotating wafold, gradually reducing its angular velocity and driving it toward a more spherical configuration. As *J* decreases, the rotational strain weakens and the polar channels become less efficient conduits for outflow, providing a geometric mechanism consistent with the observed connection between spin and jet strength.

This contrasts with other proposals for black-hole interiors. In gravastar scenarios, jet formation depends sensitively on particular matter equations of state and finely tuned shell geometries. In fuzzball models, the immense microscopic structure must somehow aggregate into a coherent macroscopic outflow. Firewall proposals posit energetic near-horizon layers but do not supply an intrinsic mechanism for large-scale collimation. Planck-star models produce significant emission only during the final explosive phase, not throughout the long-term spin evolution of an astrophysical object. By comparison, the wafold picture provides a conceptually simple geometrical explanation: a rotating terminal surface whose polar regions constitute natural channels for the release of energy and angular momentum. No exotic matter content, microstate organization, or quantum tunneling processes are required; the collimation follows directly from the geometry of a rotating, curvature-saturated boundary.

In this sense, rotating wafolds offer a new theoretical perspective on the observed link between black-hole spin and relativistic jet formation. All rotational, thermodynamic, and semiclassical fluxes are processed by the terminal surface W, which acts simultaneously as the absorber of inward semiclassical flux, the regulator of curvature, and the geometric engine for polar outflow.

### 2.6. Causal Character of the Wafold

While energy and radiation escape through the polar regions, the wafold may continue to accrete mass and angular momentum through its equatorial zones. These concurrent inflow and outflow processes shape the local causal behavior of the surface, producing regions that alternately absorb or radiate.

Therefore, the causal character of W (space-like, null, or time-like) may vary from point to point depending on the local balance between accreting and radiative fluxes. Regions dominated by inflow tend to behave in a time-like way, while highly radiative or evaporating regions may approach a null or even space-like character. Despite these local differences, the wafold’s global structure remains constrained by its conserved parameters (mass, charge, and rotation) which fix its equilibrium geometry. It cannot deform arbitrarily or grow asymmetrically into a disk after strong equatorial accretion and jet ejection. The evolution of W is therefore governed not by its local causal variations but by the net integrated flux across its surface; this global balance (whatever its sign) determines whether the wafold expands, contracts, or remains stationary, while preserving its overall topology and identity. In this sense, the wafold acts as a self-consistent geometric boundary whose shape continuously reorganizes to accommodate any pattern of inflow or outflow while preserving its overall form and stability.

The instantaneous causal character of W can be expressed through the effective normal energy density across it:

$$\Phi =\int_{W}T_{\mu \nu }^{\left(+\right)}n^{\mu }n^{\nu }dA,$$

with Φ>0 corresponding to net accretion and Φ<0 to net evaporation. Then, at a given time,

$$\varepsilon =\left\{\begin{array}{cc} -1, & \Phi >0\;(\mathrm{net}\;\mathrm{accretion},\;\mathrm{wafold}\;\mathrm{expands},\;\mathrm{space}\text{-}\mathrm{like}\;\mathrm{wafold},\;\mathrm{time}\text{-}\mathrm{like}\;n^{\mu }),\\ 0, & \Phi =0\;(\mathrm{equilibrium},\;\mathrm{null}\;\mathrm{wafold}),\\ +1, & \Phi <0\;(\mathrm{net}\;\mathrm{evaporation},\;\mathrm{wafold}\;\mathrm{retreats},\;\mathrm{time}\text{-}\mathrm{like}\;\mathrm{wafold},\;\mathrm{space}\text{-}\mathrm{like}\;n^{\mu }). \end{array}\right.$$

Under the null energy condition, the first two cases dominate: the wafold is space-like while growing and null when stationary [54]. Time-like behavior requires effective NEC violation, such as through negative-energy flux associated with Hawking evaporation [55,56].

### 2.7. Geodesic Termination

A natural question raised by the wafold hypothesis concerns the behavior of infalling trajectories, whether followed by particles, light rays, or idealized observers. In classical general relativity, such geodesics continue inward until they either extend indefinitely or encounter a curvature singularity, where the geometric description itself breaks down. In the present framework, the situation is different: the curvature does not diverge, but the spacetime manifold is taken to end at the limiting curvature hypersurface W. As an observer approaches this surface, the exterior geometry remains smooth and physical quantities along the trajectory remain finite. What ceases to exist is not the geodesic equation itself, but the manifold through which the trajectory would otherwise continue. Once the locus $K=K_{c}$ is reached, there is no further spacetime in which the affine parameter can be extended.

What occurs at the moment the trajectory intersects W is not specified here, since a full account would require a detailed understanding of how physical fields, tidal deformations, and geodesic congruences project onto or interact with the wafold. It is conceivable that certain degrees of freedom might admit an effective continuation along directions intrinsic to W, but such possibilities depend on the precise boundary dynamics and are beyond the scope of this initial construction.

## 3. Dimensional Conversion

### 3.1. Conservation

When the curvature of spacetime grows beyond a critical threshold, the present theory suggests that the three-dimensional volumetric space undergoes a conversion into a thin hypersurface, i.e., a redistribution of spatial capacity rather than a literal reduction in spacetime dimensionality. However, such a process must obey a corresponding conservation principle; otherwise, it would entail the creation or destruction of space as the black hole expands or contracts. We therefore argue that the compression of the surrounding spatial domain ingested by the wafold, denoted I, obeys a conservation law of spatial capacity. Accordingly, the absorbed volume is not lost but re-expressed as surface area when the curvature reaches a critical threshold, and shall revert if the wafold shrinks. For clarity, I refers to the spatial region that is ingested by the wafold during expansion and restored as it retracts.

Schematically, this can be written as a curvature-driven map:

(13) $$I\overset{K\to K_{c}}{\to }W.$$

The conversion (13) is not an abrupt “tearing” of space but a geometric reallocation of degrees of freedom onto the wafold. The map should conserve a global spatial capacity C, which we define as

(14) $$C=\int_{I}f\left(K_{ab}^{\left(+\right)}\right)dV+\int_{W}dA=\mathrm{const}.$$

Here, $f(K_{ab}^{\left(+\right)})$ transforms volumetric capacity into areal capacity, acting as the geometric conversion factor between the volume of I and the surface of the wafold W. Its dependence on the projected extrinsic curvature $K_{ab}^{\left(+\right)}$ reflects the fact that the conversion process is controlled by the local shape of the wafold rather than by bulk curvature invariants.

Before the curvature exceeds the critical threshold $K_{c}$, *f* has no physical meaning, as no dimensional conversion has yet begun. Once the wafold forms at $K=K_{c}$, *f* becomes a continuous function of curvature, describing how spatial capacity is redistributed across the surface. In a Schwarzschild black hole, one expects *f* to scale with the inverse curvature radius. In Section Spherical Wafold Growth, we show that, for a moving spherical wafold, conservation of C fixes *f* at $2/r_{W}$. More general functional forms of $f(K_{ab}^{\left(+\right)})$ are left for future work.

Several approaches to quantum gravity suggest that spacetime dimensionality may decrease under extreme curvature, which further motivates this idea of a geometric compression. For example, in causal dynamical triangulations, asymptotically-safe gravity, Hořava–Lifshitz models, and loop-quantum gravity, the effective dimensionality of spacetime decreases from four at macroscopic scales to about two near the Planck regime [39,57,58,59,60]. Similar behavior appears in approaches where metric degeneracy effectively removes directions at singularities [61]. These results indicate that the dimensional reduction is a generic feature of high-curvature regimes, providing physical motivation for interpreting collapse as a curvature-driven conversion of spatial volume into area.

At the mathematical level, the closest analogues to a dimensional-capacity law arise in classical geometry. The Hadamard variational formula and shape-derivative theory relate time variations of enclosed volume and surface area through curvature and normal velocity [62]. The Weyl tube and Steiner formulas link boundary area and integrated curvature to the volume of neighboring domains [63]. In general relativity, the Hawking area theorem and black-hole monotonicity laws [64] express irreversible growth in horizon area but not its conservation. Thus, while existing frameworks describe dimensional reduction or monotone evolution, none provides a conservation of dimensional capacity in which volumetric measure is continuously converted into surface measure, as proposed here.

#### Spherical Wafold Growth

To see explicitly how the dimensional conversion law operates in a simple setting, we consider a spherically symmetric Schwarzschild wafold. From Section 2.4, it follows that

(15) $$\frac{dr_{W}}{dM}=\frac{1}{3}\frac{r_{W}}{M}.$$

As an additional mass element, dM is accreted and the wafold advances from $r_{W}$ to $r_{W}+dr_{W}$, thereby absorbing a thin spherical shell of three-dimensional space. The differential volume of this shell is $dV=4\pi r_{W}^{2}dr_{W}$, while the wafold area increases by $dA=8\pi r_{W}dr_{W}$. Their ratio is

(16) $$\frac{dV}{dA}=\frac{4\pi r_{W}^{2}dr_{W}}{8\pi r_{W}dr_{W}}=\frac{r_{W}}{2}.$$

Applying the conservation law (14) to this infinitesimal step and restricting attention to the collar region swept by the wafold, the change in the volumetric contribution and the change in the surface contribution must cancel. Taking absolute variations for clarity, one has

(17) $$f(K_{ab}^{\left(+\right)})dV=dA.$$

Using (16) then fixes the conversion factor at the threshold,

(18) $$f\left(K_{ab}^{\left(+\right)}\right)=\frac{dA}{dV}=\frac{2}{r_{W}}.$$

Thus, in the spherically symmetric case, the loss of volumetric capacity of the ingested shell is exactly compensated by an increase in surface capacity on W, with $f\propto 1/r_{W}$, as anticipated.

### 3.2. Differential Form and Dimensional Flux

Differentiating the conservation relation (14) gives

(19) $$\frac{dC}{dt}=\frac{d}{dt}\left(\int_{I(t)}f\left(t\right)dV+\int_{W(t)}dA\right)=0.$$

Again, we interpret I as a collar region located just outside the wafold, bounded internally by the wafold surface W(t) and externally by an outer surface Γ. The conversion process occurs from the inside out: as the wafold expands, it absorbs the spatial volume within this collar. Therefore, the outer boundary Γ marks the limit of the region affected by the conversion and remains fixed in space; its normal velocity is zero, and any contribution from it vanishes. The only nonvanishing contribution of ∂I comes from the wafold itself, ∂I≡W.

Applying the Reynolds transport theorem to a quantity f(R) defined in I(t) gives

(20) $$\frac{d}{dt}\int_{I(t)}fdV=\int_{I}\partial_{t}fdV+\underset{=0}{\underset{︸}{\int_{\Gamma }fv_{n}{|}_{\Gamma }dA}}-\int_{W}fv_{n}{|}_{W}dA,$$

where $v_{n}{|}_{\Gamma }$ and $v_{n}{|}_{W}$ denote the normal velocities of the outer and inner boundaries, respectively. Since the outer surface Γ is fixed, $v_{n}{|}_{\Gamma }=0$, and only the wafold contributes, $v_{n}{|}_{W}=v_{n}$, with $v_{n}$ measured along the unit normal n pointing from W outward into I. Here, $v_{n}>0$ corresponds to outward expansion of the wafold.

Next, the Hadamard formula expresses the variation in a surface area integral when the surface W(t) moves normally with velocity $v_{n}$.

(21) $$\frac{d}{dt}\int_{W(t)}dA=\int_{W}Hv_{n}dA,$$

where H=∇·n is the mean curvature of W. Positive *H* corresponds to convex surfaces expanding outward. Combining (20) and (21) into the total derivative of C and rearranging terms yields

(22) $$\int_{I}\partial_{t}fdV=\int_{W}\left(f-H\right)v_{n}dA.$$

Equation (22) thus represents the global balance between the bulk variation in spatial capacity and the net conversion “flux” across the wafold.

#### 3.2.1. Dimensional Continuity and Flux Form

Equation (22) can be recast in divergence form, if we define the dimensional flux vector as

(23) $$J_{D}=\left(f-H\right)v_{n}n,$$

whose divergence gives the local rate of change in the volumetric capacity density field:

(24) $$\nabla \cdot J_{D}=\partial_{t}f.$$

This could be seen as a *dimensional continuity law*: the divergence of the dimensional flux equals the temporal variation in the spatial capacity.

(25) $$\int_{I}\left(\nabla \cdot J_{D}\right)dV=\int_{W}J_{D}\cdot ndA.$$

Here, $J_{D}$ acts as a geometric current transferring capacity from the three-dimensional exterior domain into the wafold.

#### 3.2.2. Stokes Representation

The relations derived above, particularly (24) and (25), naturally evoke Stokes’ theorem: a general statement that the integral of a divergence over a volume equals the flux of the corresponding quantity through its boundary. In the present context, this means that the net conversion of volumetric capacity within I is exactly represented by the flux of the dimensional current through the wafold W. In differential-form language, this conservation relation can be written compactly as

(26) $$d\beta =\left(\partial_{t}f\right){\mathrm{vol}}_{3},\;\;\beta =\left(f-H\right)v_{n}\iota_{n}{\mathrm{vol}}_{3},$$

where ${\mathrm{vol}}_{3}$ is the spatial volume form and $\iota_{n}$ denotes contraction along the normal vector. This formulation expresses the same continuity law as (24) but in exterior-calculus form, making explicit that the dimensional current is a conserved geometric flux whose exterior derivative represents the local source converting volume into area. By Stokes’ theorem, $\int_{I}d\beta =\int_{\partial I}\beta ,$ one recovers the global balance relation (22). Equation (24) thus characterizes a conserved *dimensional current*: the exterior derivative of the flux form equals the local source term that converts volumetric capacity into areal geometry.

#### 3.2.3. Physical Interpretation

The vector $J_{D}$ represents a *dimensional flux*: a geometric current that quantifies how volumetric capacity is converted into areal capacity as the wafold moves. Its divergence governs the local rate at which three-dimensional volume is re-expressed as surface geometry, according to the continuity relation (24). In this sense, C functions as a conserved “dimensional charge” whose flow across W implements the conversion process. The two factors appearing in $J_{D}$ play distinct geometric roles. The term (f−H) acts as the *local driving potential*. It measures the mismatch between the conversion factor *f*, which encodes how much surface area must replace a given volume element, and the mean curvature *H*, which controls how the area of the moving surface changes as it evolves. The normal velocity $v_{n}$ provides the kinematic component of the flux: only motion of the wafold relative to the exterior domain transports dimensional capacity. Different regimes follow directly from this structure. When f>H, the conversion of an ingested volume element produces more area than required by the mean-curvature geometry of the surface, yielding a positive flux $\left(J_{D}\cdot n>0\right)$; the wafold behaves as a *source* of dimensional flux. When f<H, the converse occurs: the conversion produces an insufficient area relative to the geometric demand of the surface, yielding a negative flux $\left(J_{D}\cdot n<0\right)$; the wafold acts as a *sink*. In the special case where f=H, the dimensional flux vanishes regardless of the surface’s motion. This corresponds to an equilibrium configuration in which the geometric change in area associated with the wafold’s evolution is exactly balanced by the conversion of the ingested volume.

For a spherically symmetric Schwarzschild wafold, this equilibrium is realized automatically: at the curvature threshold, we saw that $f=2/r_{W}$, while the mean curvature of a round sphere is also $H=2/r_{W}$, giving $J_{D}=0$. In this case, the vanishing of $J_{D}$ does not imply that the wafold is static. Even if the surface moves outward or inward ($v_{n}\neq 0$), the conversion of the ingested volume produces exactly the amount of new area required by the geometry of a sphere of radius $r_{W}\left(t\right)$. The wafold therefore changes size without generating any net dimensional flux: its evolution remains perfectly balanced, and the spherical surface acts as a geometric fixed point of the conversion law.

Away from spherical symmetry (such as in rotating, accreting, or dynamically evolving configurations), *f* and *H* generally differ, and $J_{D}$ provides a covariant measure of the resulting geometric departure from this equilibrium.

## 4. Entropy, Information, and Conservation

### 4.1. Area Scaling from Confinement

If the degrees of freedom associated with a collapsing configuration are confined to the terminating hypersurface W, then the number of distinguishable microstates is naturally expected to scale with the area of that surface rather than with any interior volume. This follows from two basic assumptions: (i) that crossing the curvature threshold causes all relevant degrees of freedom to migrate to the terminating hypersurface, and (ii) a finite information density per unit area, plausibly of order one bit per Planck area. Under these conditions, the entropy takes the generic form

(27) $$S\propto A_{W},$$

providing a geometric explanation for why black-hole entropy should be an area quantity rather than a volumetric one.

This helps clarify the tension in classical reasoning: ordinary matter exhibits volumetric entropy because its microscopic configurations occupy a three-dimensional domain, whereas black holes exhibit area scaling as expressed in the Bekenstein–Hawking relation,

(28) $$S_{\mathrm{BH}}=\frac{k_{B}A_{H}}{4L_{P}^{2}}.$$

Within the present framework, this difference arises because the relevant degrees of freedom are confined to a surface produced by curvature-driven compression. The entropy therefore scales with the size of this surface rather than with any interior volume.

However, the wafold hypothesis by itself does not determine the normalization of the entropy. In general, the wafold area $A_{W}$ is not equal to the horizon area $A_{H}$; for astrophysical black holes, one typically has $A_{W}\ll A_{H}$. In certain regimes (e.g., near-Planckian masses or in highly dynamical collapse), the two surfaces may approach one another or even coincide, but this is not necessarily generic. The numerical coefficient 1/4 in the Bekenstein–Hawking formula ultimately arises from semiclassical considerations tied to the exterior geometry and to Hawking radiation, rather than from the geometry of the wafold itself.

In this sense, the present framework plays a complementary role: the wafold provides a geometric rationale for the scaling S∝A, while the semiclassical physics of the exterior spacetime fixes the specific normalization $S=A_{H}/4$. This situation is analogous to other surface-based descriptions (such as the membrane paradigm, fuzzball constructions, and gravastar models), in which microstates reside on a surface that need not coincide with the event horizon, yet the thermodynamic entropy remains governed by the horizon area. Accordingly, we regard the wafold picture as offering a geometric underpinning for the area law without claiming a microscopic derivation of the Bekenstein–Hawking prefactor.

### 4.2. Information Conservation During the Conversion

As the wafold expands and ingests mass–energy, there can be no loss of information in the volume-to-area conversion. All information previously encoded within the volume now bound by the wafold, which we denote by U, must be transferred onto W. Let $\rho_{U}$ denote the volumetric information density that existed in U and $\sigma_{U}$ the corresponding surface density on W. Information conservation then requires

(29) $$\int_{U}\rho_{U}dV=\int_{W}\sigma_{U}dA,$$

ensuring that the total informational content of the system remains invariant even though its geometric support changes from a volume *V* to an area *A*.

In this framework, information is not destroyed or radiated into a hidden region but geometrically reorganized. Since the interior is no longer part of physical reality, there is no domain in which information could be lost: it is preserved on the hypersurface that replaces it. This provides a geometric resolution to the black-hole information problem without invoking microscopic dynamics beyond W [29,65].

From an informational standpoint, the wafold serves as a record of the system’s past: every degree of freedom that would have entered the interior is re-expressed as a configuration on W. The process conserves total information while merely changing the form of its representation (from a distributed volumetric encoding to a confined surface encoding).

In (29), we implicitly assume a closed conversion process, in which no net information flux enters or leaves U during the transformation, so that all information initially associated with the volume becomes encoded on W. The general situation, in which information may be exchanged with the exterior during the conversion (for example through neighboring infalling matter during accretion), can be incorporated by adding explicit flux terms, leading to a more generalized formula that we will discuss in Section 4.4, analogous to the dimensional-flux conservation law (22).

### 4.3. Semiclassical Information Flow: Hawking Radiation as Tunneling

Beyond the geometric conservation encoded by the wafold, semiclassical analyses show that Hawking radiation itself carries correlations capable of preserving information. In the Parikh–Wilczek tunneling picture [66,67,68,69,70], energy conservation and backreaction slightly deform the emission spectrum away from perfect thermality. Each emitted quantum reduces the mass of the black hole, and this induces correlations among successive emissions. These correlations imply that Hawking radiation can, in principle, encode partial information about the collapsing state at the semiclassical level, even without invoking a full quantum-gravity description.

This is compatible with the wafold framework. The wafold does not modify the standard exterior mechanism that produces Hawking radiation: the semiclassical tunneling picture remains unchanged outside the curvature-defined surface. What changes is only the fate of infalling degrees of freedom, which no longer propagate into an ill-defined interior but instead terminate on the hypersurface W.

In this combined picture, the wafold acts as the geometric repository for the information that semiclassical tunneling gradually imprints into the outgoing Hawking flux. Standard tunneling analyses assume that the information driving backreaction resides somewhere inside the black hole, yet the classical interior ends at a singularity, leaving no clear locus for storing or interacting with this information. By replacing the singularity with a finite, curvature-defined hypersurface that accumulates all infalling degrees of freedom, the wafold provides the explicit structure required for backreaction-induced correlations to originate. The geometric confinement of information on W and the semiclassical transfer of information through correlated radiation therefore address complementary aspects of the information problem. A full proof of unitarity would still require a complete microscopic theory, but semiclassical tunneling already shows that information is not automatically lost once the interior is replaced by a well-defined terminal surface.

### 4.4. Generalized Boundary Projection of Fluxes

Up to this point, the discussion has focused on information: how it is stored on W and how semiclassical correlations in Hawking radiation can transmit part of it outward. We now turn to the general geometric structure that governs all conserved currents. The mechanism that projects informational flux onto W applies equally to any physical current defined in the manifold.

In Section 3, the dimensional conversion law established that a bulk divergence $\nabla \cdot J_{D}$ is balanced by a surface flux across W. The same geometric relation holds for any conserved current J, whether it represents energy, momentum, charge, or information.

For any smooth current J defined in the physical domain M whose boundary is the wafold, ∂M≡W, the integral relation reads

(30) $$\int_{M}\left(\nabla \cdot J\right)dV=\int_{W}J\cdot n\;dA,$$

where n is the outward unit normal to W. No further introduction of Stokes’ theorem is necessary here: Equation (30) is its direct geometric expression in the notation already introduced in Section 3.

In this generalized sense, the wafold provides a universal geometric projection in which any flux that would have propagated into the interior is mapped onto W as a surface density. Energy fluxes ($J_{E}$), charge fluxes ($J_{Q}$), informational fluxes ($J_{I}$), and as we define them in this theory, geometric-capacity fluxes ($J_{D}$), all share this property.

In summary, the termination of the manifold converts all bulk conservation statements into boundary ones. Here, the wafold stands as the final geometric ledger, and every conserved quantity finds its surface expression.

## 5. Physical Consistency

Any geometric reinterpretation of black holes must remain compatible with well-tested exterior physics, with the causal structure of general relativity, and with the semiclassical behavior of quantum fields. In this section, we gather these requirements in one place and provide a minimal sketch of consistency checks for the wafold construction.

### 5.1. Minimal Consistency Checks

The first two basic conditions are as follows:

1. Exterior invariance: The external spacetime must remain effectively indistinguishable from the classical Schwarzschild or Kerr solution. The proposed dimensional conversion occurs only at or inside the wafold W, and does not alter the vacuum Einstein equations in the exterior domain. All observables derived from the metric outside W (including light deflection, orbital precession, redshift, and gravitational-wave emission) are expected to coincide with those of standard general relativity to within current experimental precision. In this sense, the wafold represents an inner geometric modification that leaves the external gravitational field intact.
2. Causal completeness: The spacetime remains globally causal; every causal curve is extendible only up to the boundary W, which replaces the singularity as the legitimate endpoint of all infalling trajectories. No geodesic continues into the non-reality region, since beyond W the manifold ceases to exist. Observers may describe infall as approaching W asymptotically, but no events beyond it belong to the spacetime.

Taken together, these conditions indicate that the dimensional conversion framework can be formulated in a manner consistent with standard exterior physics and with the causal structure of general relativity, at least at the level of the preliminary arguments presented here. The wafold modifies only an interior region inside the event horizon, while preserving the empirical and mathematical structure of general relativity in all other domains.

### 5.2. Semiclassical Backreaction in the Presence of a Wafold

In semiclassical gravity, the exterior metric evolves according to

(31) $$G_{\mu \nu }\left[g^{\left(+\right)}\right]=8\pi {\langle T_{\mu \nu }\rangle }_{\mathrm{QFT}},$$

where $g^{\left(+\right)}$ is the exterior geometry and ${\langle T_{\mu \nu }\rangle }_{\mathrm{QFT}}$ is the expected (renormalized) stress tensor of quantum fields defined on that geometry [71,72,73]. Since the wafold removes only the manifold region $r<r_{W}$, Equation (31) continues to govern the dynamics for all $r>r_{W}$. The nontrivial question is how ${\langle T_{\mu \nu }\rangle }_{\mathrm{QFT}}$ behaves in the vicinity of a one-sided geometric boundary. In the wafold framework, the boundary W is not merely a mathematical cutoff for field propagation, but a genuine geometric endpoint that absorbs all fluxes which would otherwise enter the classical interior. In particular, the inward negative-energy flux associated with Hawking radiation is deposited directly onto W, contributing to its surface stress–energy and driving its slow inward motion through the curvature-threshold condition. From the semiclassical perspective, this means that ${\langle T_{\mu \nu }\rangle }_{\mathrm{QFT}}$ need only be defined in the exterior domain, with its normal projections onto W encoding the exchange of energy and momentum between quantum fields and the terminal surface.

In related settings, such as end-of-the-world branes in AdS/BCFT constructions, quantum fields can be defined consistently on spacetimes that terminate on a codimension-one hypersurface, provided suitable geometric boundary conditions (Dirichlet, Neumann, or mixed) are imposed. In those cases, the renormalized stress tensor remains finite and is also determined entirely by the exterior geometry and the chosen boundary conditions. By analogy, we assume that an appropriate choice of boundary condition on the wafold W exists such that the renormalized stress tensor ${\langle T_{\mu \nu }\rangle }_{\mathrm{QFT}}$ can be understood solely as an exterior limit, with boundary data on W accounting for the absorption of fluxes that would otherwise propagate into an interior continuation. A detailed construction of this boundary QFT is beyond the scope of the present work, but there is no obvious obstruction at the level of semiclassical geometry [74].

Under this assumption, the semiclassical backreaction outside the wafold is governed by the same equations as in ordinary evaporating black holes. The curvature threshold modifies only the region that has been excised; the quantum state of the exterior fields, and hence the Hawking flux and its backreaction on $g_{\mu \nu }^{\left(+\right)}$, can be treated in the usual way.

### 5.3. Hawking Flux

Hawking radiation is accompanied by a compensating inward flux of negative energy, required by energy conservation and encoded in ${\langle T_{\mu \nu }\rangle }_{\mathrm{QFT}}$. In the classical picture, this flux is absorbed near the singularity; in the wafold scenario, it is instead absorbed directly by the terminal surface W. The normal projection $T_{\mu \nu }^{\left(+\right)}n^{\mu }n^{\nu }$ therefore contributes to an effective inward pressure on W, leading to a slow decrease in its radius. A schematic energy balance suggests a relation of the form

(32) $${\dot{r}}_{W}\propto -T_{\mu \nu }^{\left(+\right)}n^{\mu }n^{\nu },$$

analogous to the inward migration of apparent horizons in evaporating black holes. We do not attempt to derive the proportionality factor, as it depends on the precise boundary conditions on W and on the full semiclassical dynamics. For present purposes, we assume that the decreasing ADM mass M(t) drives the wafold radius through the curvature-threshold condition,

(33) $$K\left(r_{W}\left(t\right);M\left(t\right)\right)=K_{c},$$

with K(r) given by Equation (3). In the quasi-stationary regime (where M(t) evolves slowly compared to the geometric timescales), the wafold simply tracks the locus of limiting curvature while the exterior remains well approximated by the semiclassically corrected Schwarzschild/Kerr geometry.

### 5.4. Stability of the Wafold Surface

A full stability analysis of the wafold requires deriving the quadratic (and higher) action governing perturbations of the terminal hypersurface W. In the present framework, W is not a junction surface but a one-sided boundary of the spacetime manifold; consequently, the appropriate starting point is the one-sided gravitational action (10). Perturbative stability is then analyzed by examining the second variation in this action under deformations of the boundary, which captures the leading-order response of the wafold to small geometric perturbations.

#### 5.4.1. Normal-Mode Parametrization

Let $X^{\mu }$ denote the spacetime position of points on the hypersurface W. A general infinitesimal deformation of W may be decomposed into tangential and normal components. Tangential deformations correspond to reparametrizations of W and are therefore pure gauge. The physical shape fluctuation is captured by a single scalar normal deformation field ψ defined on W, which generates an infinitesimal displacement

(34) $$\delta X^{\mu }=\psi \;n^{\mu },$$

where $n^{\mu }$ is the outward unit normal to W. Positive values of ψ correspond to displacements along the outward normal, while negative values correspond to inward deformations of the surface.

#### 5.4.2. Quadratic Action from the One-Sided Variational Principle

Consider the one-sided action

(35) $$S\left[g\right]=\frac{1}{16\pi }\int_{M}R\sqrt{-g}d^{4}x+\frac{1}{8\pi }\int_{W}K^{\left(+\right)}\sqrt{\left|h\right|}d^{3}x+S_{\mathrm{ext}},$$

with ∂M≡W. When the exterior spacetime satisfies the vacuum Einstein equations ($R_{\mu \nu }=0$ away from sources), the bulk term makes no contribution to the dynamics of small deformations. As a result, the leading response to perturbations of the terminal surface W is entirely controlled by the boundary term and its geometric variation under normal displacements (34). The resulting quadratic effective action for the normal deformation mode takes the universal Jacobi form

(36) $$S_{W}^{\left(2\right)}\left[\psi \right]=\frac{1}{16\pi }\int_{W}\sqrt{\left|h\right|}\psi L_{W}\psi d^{3}x,$$

where $L_{W}$ is a second-order self-adjoint differential operator acting on the scalar deformation field ψ defined on W.

A convenient covariant expression for the $L_{W}$ operator is

(37) $$L_{W}=-\varepsilon \Delta_{W}-\left({\mathrm{Ric}}_{\mu \nu }^{\left(+\right)}n^{\mu }n^{\nu }+K_{ab}^{\left(+\right)}K_{\left(+\right)}^{ab}\right)+V_{\mathrm{bc}},$$

with $\Delta_{W}=h^{ab}D_{a}D_{b}$, the Laplace–Beltrami operator on W, and $D_{a}$ as the Levi–Civita connection of the induced metric $h_{ab}$. The term ${\mathrm{Ric}}_{\mu \nu }^{\left(+\right)}n^{\mu }n^{\nu }$ probes the curvature of the exterior spacetime along the normal direction and it vanishes in exterior vacuum but is retained here for generality. The quantity $K_{ab}^{\left(+\right)}K_{\left(+\right)}^{ab}$ is nonnegative and acts as a geometric “mass” term that penalizes shape fluctuations of the surface. The contribution $V_{\mathrm{bc}}$ encodes additional boundary physics associated with the implementation of the terminal condition at W (for example, whether the defining condition is enforced sharply or via a boundary potential fixing $K=K_{c}$). In the minimal geometric picture adopted here, $V_{\mathrm{bc}}$ is dominated by the curvature-threshold enforcement discussed below.

Equation (36) makes the stability criterion transparent. Expanding the deformation in eigenmodes $\psi =\sum_{i}c_{i}\psi_{i}$ satisfying $L_{W}\psi_{i}=\lambda_{i}\psi_{i}$, the sign of the eigenvalues $\lambda_{i}$ determines whether small perturbations increase or decrease the action. For a space-like normal (ε=+1), the gradient term contributes positively, while for a time-like normal (ε=−1), the kinetic signature is reversed, in direct correspondence with the causal character of W.

#### 5.4.3. Implementing the Limiting-Curvature Condition as a Constraint

One way to incorporate the invariant condition $K=K_{c}$ into the fluctuation analysis is to treat it as a boundary constraint enforced by a Lagrange multiplier field λ on W,

(38) $$S_{\mathrm{constr}}=\int_{W}\sqrt{\left|h\right|}\lambda \left(K-K_{c}\right)d^{3}x.$$

Normal deformations move the surface to a nearby embedding,

$$X^{\mu }\to X^{\mu }+\psi n^{\mu }.$$

Accordingly, expanding the extrinsic curvature under a normal deformation yields

(39) $$K\left(X+\psi n\right)=K_{0}+\delta K\left[\psi \right]+\frac{1}{2}\delta^{2}K\left[\psi ,\psi \right]+\cdots ,\;\;K_{0}=K_{c}.$$

Since the undeformed surface already satisfies the condition $K_{0}=K_{c}$, the zeroth-order term vanishes. Varying with respect to the Lagrange multiplier then enforces the linearized condition δK[ψ]=0, so no linear term is generated in the action. Instead, the allowed normal deformations are restricted to those that leave the extrinsic curvature unchanged at the first order. Deformations that violate this condition would immediately move the surface away from the limiting-curvature locus and are therefore excluded. As a consequence, the first nontrivial effect of the constraint appears at the quadratic order, through higher variations such as $\delta^{2}K$. At the level of the quadratic theory considered here, the effect of the curvature condition is to exclude deformation directions that would violate $K=K_{c}$.

#### 5.4.4. Schwarzschild Specialization

In an exact Schwarzschild exterior, the Kretschmann scalar depends only on the radius, $K\left(r\right)=48M^{2}/r^{6}$, and is strictly monotonic. The unperturbed wafold radius $r_{W}^{\left(0\right)}$ is therefore uniquely fixed by the curvature-threshold condition $K\left(r_{W}^{\left(0\right)}\right)=K_{c}$.

To connect with the general fluctuation framework above, we now give a direct geometric argument showing how this condition constrains small deformations of the wafold in the Schwarzschild case. Consider a perturbed wafold surface,

(40) $$r_{W}\left(t,\theta ,\phi \right)=r_{W}^{\left(0\right)}+\delta r\left(t,\theta ,\phi \right).$$

Because the wafold is defined by saturation of the curvature bound, admissible perturbations must preserve this condition,

(41) $$K\left(r_{W}^{\left(0\right)}+\delta r\right)=K_{c}=K\left(r_{W}^{\left(0\right)}\right).$$

Subtracting the unperturbed relation yields the consistency requirement

(42) $$\delta K\equiv K\left(r_{W}^{\left(0\right)}+\delta r\right)-K\left(r_{W}^{\left(0\right)}\right)=0.$$

Expanding to the first order gives

(43) $$\delta K=\frac{\partial K}{\partial r}{|}_{r_{W}^{\left(0\right)}}\delta r+\delta K_{\mathrm{ang}}=0,$$

where $\delta K_{\mathrm{ang}}$ denotes the curvature change induced by angular distortions of the surface. Equation (43) simply states that the perturbed surface must remain on the locus $K=K_{c}$.

If the deformation is spherically symmetric, the angular contribution vanishes and one has

$$\delta K=\frac{\partial K}{\partial r}{|}_{r_{W}^{\left(0\right)}}\delta r.$$

For Schwarzschild,

$$\frac{\partial K}{\partial r}=-\frac{288M^{2}}{r^{7}}<0.$$

Thus, any outward displacement decreases *K*, moving the surface below the threshold, while any inward displacement attempts to raise *K* above $K_{c}$, which is forbidden by the limiting-curvature condition. In this quasi-static sense, the monotonicity of K(r) selects a unique allowed radius and acts as a geometric restoring mechanism for radial perturbations.

For nonspherical perturbations, the curvature variation includes the angular term $\delta K_{\mathrm{ang}}$ arising from distortions of the spherical shape. In an exact Schwarzschild geometry, however, *K* carries no angular dependence. As a result, $\delta K_{\mathrm{ang}}$ cannot be compensated independently: angular deformations are necessarily coupled to a radial shift δr through (43) and are therefore likewise constrained by the curvature-threshold condition. In this sense, nonspherical distortions of W are also restricted and cannot grow freely.

Taken together, these considerations show that in an exact Schwarzschild exterior, the wafold is not merely linearly stable but geometrically rigid under the defining condition $K=K_{c}$. All admissible linear deformations are eliminated, and the remaining dynamics reduces to the evolution of the single collective degree of freedom $r_{W}\left(t\right)$, driven by the net exterior fluxes discussed in Section 5.

### 5.5. Summary of Consistency Checks

The points above suggest that the wafold construction can be made broadly compatible with established aspects of black-hole physics, at least at a preliminary level. A concise summary is as follows:

- Exterior invariance. All geometric modifications occur only for $r<r_{W}$. The exterior spacetime continues to satisfy the vacuum Einstein equations and remains observationally consistent with the Schwarzschild/Kerr solution across current empirical tests.
- Causal completeness. The surface W supplies a well-defined endpoint for infalling trajectories. Causal curves do not extend beyond this hypersurface, not because of divergent curvature but because the manifold itself terminates there.
- Semiclassical backreaction. Since the exterior region is unchanged, the semiclassical equation
  $$G_{\mu \nu }\left[g^{\left(+\right)}\right]=8\pi {\langle T_{\mu \nu }\rangle }_{\mathrm{QFT}}$$
  continues to govern the evolution for all $r>r_{W}$. Quantum fields may be defined on a spacetime with a one-sided boundary, provided suitable boundary conditions are imposed, and the renormalized stress tensor is determined entirely by the exterior geometry.
- Hawking flux and evolution of the wafold. The negative-energy flux associated with Hawking radiation is absorbed directly by W, leading to a gradual decrease in its radius. In a quasi-stationary regime, the wafold radius tracks the condition
  $$K\left(r_{W}\left(t\right);M\left(t\right)\right)=K_{c},$$
  mirroring the slow mass loss of the black hole.
- Stability. Perturbations of W are constrained by the requirement that the surface continues to satisfy $K=K_{c}$. For spherical deformations, the monotonicity of K(r) selects a unique radius, while nonspherical deformations are tied to the radial shift through the same constraint. This coupling limits the ability of angular distortions to grow unchecked.

These considerations indicate that the wafold hypothesis can satisfy several basic consistency requirements and does not obviously conflict with established semiclassical or geometric principles.

## 6. Limitations and Clarifications

### 6.1. What This Model Does Not Claim

We do not propose a microphysical substrate, quantize gravity, or offer a complete action principle. We avoid speculating about cosmology or multiverse extensions. Our aim is geometric clarity: define the boundary, articulate conservation and causality, and prove that a surface description suffices to remove singularities while preserving exterior physics.

### 6.2. Minimal Assumptions

Within the minimal framework adopted here (classical general relativity outside the wafold, spherical symmetry, a universal curvature bound, and a fixed Schwarzschild exterior mass), mass–energy cannot be supported at radii smaller than the limiting-curvature scale. Under these conditions, a shell-like concentration of mass–energy at the radius where the curvature bound is saturated appears to be the most straightforward configuration compatible with both the curvature limit and a nonzero ADM mass. In this restricted sense, the wafold and its associated dimensional compression do not constitute additional assumptions, but rather arise as a natural geometric outcome of imposing these constraints. More elaborate scenarios (including nonspherical collapse, exotic matter, or full quantum-gravity dynamics) may permit alternative interior behaviors, but such possibilities require relaxing one or more of the minimal assumptions considered here.

### 6.3. Type of Black Holes Considered

Throughout this paper, we restrict attention to classical, vacuum black holes described by the Schwarzschild and Kerr families of general relativity, i.e., asymptotically flat, stationary solutions with or without angular momentum, and with negligible charge. These idealized geometries capture the essential features of astrophysical black holes once external accretion flows are neglected, and they provide the cleanest setting in which to discuss geometric termination. The wafold hypothesis is therefore formulated in this minimal context, but in principle the same reasoning could extend to charged (Reissner–Nordström) or cosmological (de Sitter) configurations if the curvature threshold and termination surface were defined covariantly. In discussing surfaces, we distinguish the wafold from global event horizons and quasi-local trapping/apparent horizons; the wafold is defined by a critical-curvature condition and may coincide with such horizons only in special circumstances (e.g., stationary limits or specific mass scales).

### 6.4. Open Mathematical Tasks

Open mathematical tasks are as follows: (i) describing how three-dimensional spatial degrees of freedom reorganize during dimensional compression; (ii) formulating the behavior of gravity within or along the wafold; (iii) deriving further the evolution equations from the exterior geometry; (iv) elaborating a case-by-case precise functional form of $f(K_{ab}^{\left(+\right)})$; (v) further clarifying how boundary conditions on the terminal hypersurface may be specified within the wafold framework; and (vi) studying the relation between the wafold and standard quasi-local horizons (apparent, trapping, and dynamical horizons).

## 7. Conclusions

We have presented the concept of the wafold, a theoretical framework in which the region inside a black hole is replaced by a curvature-triggered terminal hypersurface. According to this, when curvature approaches a critical limit, spatial dimensionality is compressed: the three-dimensional space manifold collapses into a closed, thin, shell-like hypersurface that confines all mass–energy and information. This offers a geometric reason for area-scaling of entropy, replaces the singularity with a causal termination, yielding a self-sustained superficial and hollow black hole whose geometry requires no internal support, and leaves the external Schwarzschild/Kerr geometry intact. The resulting model is conceptually radical: it preserves causality and information without invoking hidden interiors, firewalls, or exotic matter. Instead, it treats the disappearance of the manifold as a geometric transition governed by a proposed *dimensional conversion law*. Interpreting black holes as points where spacetime reaches its limit, this work elaborates on a geometric alternative to the singularity, to be further developed through theoretical and numerical investigation. Even so, it frames a simple possibility: that the limit of spacetime is not concealed within a black-hole singularity, but expressed on a surface.

## Figures and Tables

**Table 1:** Radial regions of the Schwarzschild geometry and the location of the wafold surface.

Region	Radial Range	Description
Asymptotic exterior	r≫2M	Weak–field Schwarzschild
Exterior region	r>2M	Regular vacuum exterior
Event horizon	r=2M	Regular null surface
Interior region	$2M>r>r_{W}$	Interior Schwarzschild vacuum (if $r_{W}<2M$)
Wafold surface	$r=r_{W}$	Curvature reaches $K_{c}$; manifold terminates
Post–wafold	$r<r_{W}$	No geometric extension

*Note.* For near-Planckian masses, the curvature threshold can be reached before a classical interior develops, in which case $r_{W}$ may approach or coincide with the event horizon at r=2M.

## Data Availability

No new data were generated or analyzed in this study. All statements are conceptual and supported by sources cited in the bibliography; therefore, no dataset is associated with this article.
